# A Plasma Cell Dyscrasia Presenting as Amyloid Cardiomyopathy and Autonomic Dysfunction in a Healthy Patient

**DOI:** 10.7759/cureus.1409

**Published:** 2017-06-29

**Authors:** Rabih Tabet, Julie Zaidan, Boutros Karam, Samer Saouma, Foad Ghavami

**Affiliations:** 1 Internal Medicine, Staten Island University Hospital, Northwell Health; 2 Cardiology, Staten Island University Hospital, Northwell Health

**Keywords:** systemic amyloidosis, amyloid cardiomyopathy, plasma cell dyscrasia, autonomic dysfunction

## Abstract

Systemic amyloidosis is a rare multisystem disease caused by incorrectly folded proteins that deposit pathologically in different tissues and organs of the human body. It has a very wide spectrum of clinical presentations according to the affected organ(s), and its diagnosis is commonly delayed. Cardiac involvement is the leading cause of morbidity and mortality and carries a poor prognosis, especially in primary light chain amyloidosis. Therefore any delay in the diagnosis can result in devastating outcomes for the patient. We report the case of a 65-year-old man who presented with dizziness and lightheadedness. He was found to have orthostatic hypotension and further investigations revealed the diagnosis of amyloid cardiomyopathy complicating a plasma cell dyscrasia. What is worth noting, in this case, is that the patient had cardiac amyloidosis presenting primarily as autonomic dysfunction and orthostatic hypotension, without any cardiac-specific symptoms such as heart failure or angina. This is a very unusual presentation of advanced-stage cardiac amyloidosis. This article highlights the variety of clinical presentations of cardiac amyloidosis, and focuses on the recent progress such as novel diagnostic and surveillance approaches using imaging, biomarkers, and new histological typing techniques. Current and future promising treatment options are also discussed, including methods directly targeting the amyloid deposits.

## Introduction

Amyloid cardiomyopathy (AC) is an uncommon medical condition resulting from a systemic disease known as amyloidosis. This disease is still widely underdiagnosed as there are several types of amyloid, each with its unique features, leading to a vast spectrum of clinical presentations. The purpose of this article is to familiarize readers with the various clinical features of amyloidosis, to address the approach toward this disease, and to focus mainly on the cardiovascular presentation, treatment options, and prognosis. Here, we describe the case of amyloid cardiomyopathy in an adult male who presented with autonomic dysfunction; he was eventually diagnosed with primary amyloidosis and myocardial involvement secondary to a plasma cell dyscrasia.

## Case presentation

**Figure 1:**
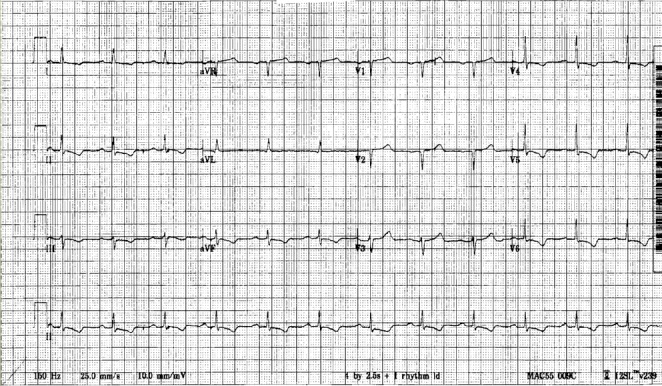
Electrocardiogram (ECG) upon admission ECG upon presentation showing microvoltage, first degree atrio-ventricular block, and poor R-wave progression on precordial leads, with infero-lateral non-specific ST-T wave changes.

A 65-year-old man was admitted to our institution for evaluation of dizziness and generalized weakness. His symptoms started two months prior with a progressive increase in dizziness described as unsteadiness and lightheadedness; it was more prominent when standing up, and improved with sitting still. He was evaluated back then, and was told that he had vertigo which was treated with meclizine. His symptoms did not get better after initiation of the treatment; on the contrary, they gradually got worse. Moreover, the patient has suffered several falls that he attributed to his dizziness. Also, he lost around forty pounds unintentionally over the last year despite preserving a good appetite. At his presentation, the patient was in no distress, complaining only of dizziness especially when trying to get up or walk. On physical examination, he had a blood pressure of 97/60 mmHg and a heart rate of 76 beats per minute while lying supine. When asked to stand up, his blood pressure dropped to 76/45 mmHg and his heart rate rose to 81 beats per minute. He had no other significant findings, in particular, neurological and cardiovascular examinations were normal. Laboratory tests came back unremarkable except for mild normocytic anemia with a hemoglobin level of 11.8 g/dL and proteinuria of 30 mg/dL. The electrocardiogram (ECG) showed micro-voltage with first-degree heart block and pseudo-infarction pattern on the precordial leads along with infero-lateral non-specific ST-T wave changes (Figure 1).

**Figure 2:**
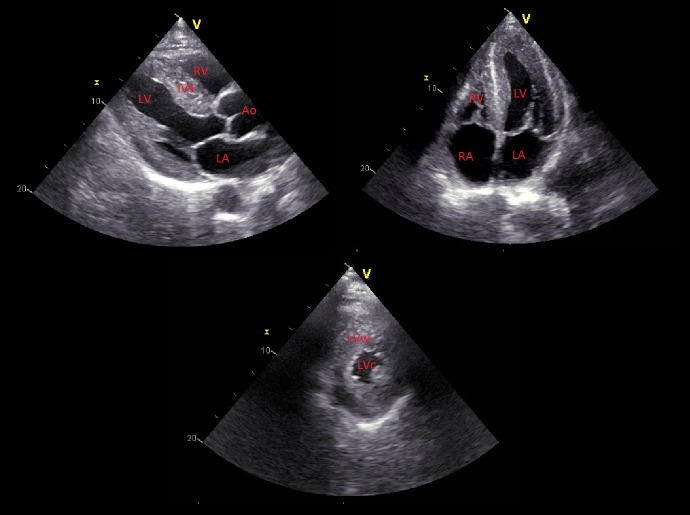
Echocardiogram Echocardiogram showing left ventricular hypertrophy and myocardial sparkling with an interventricular septum of 16 mm, a small left ventricular cavity with bi-atrial enlargement. LV: Left ventricle; RV: Right ventricle; LA: Left atrium; RA: Right atrium; Ao: Aorta; IVS: Interventricular septum; LVc: Left ventricular cavity; LVw: Left ventricular wall.

A chest x-ray, computed tomography (CT) scan of the head, and a duplex scan of the carotids failed to explain the patient’s symptoms. Transthoracic echocardiography (TTE) revealed a left ventricular hypertrophy with an inter-ventricular septal thickness of 16 mm along with a reduced systolic function and an ejection fraction around 45% (Figure 2). The myocardium had a speckled/granular appearance with increased echogenicity, and there was mild homogenous thickening of the mitral and tricuspid valves.

**Figure 3:**
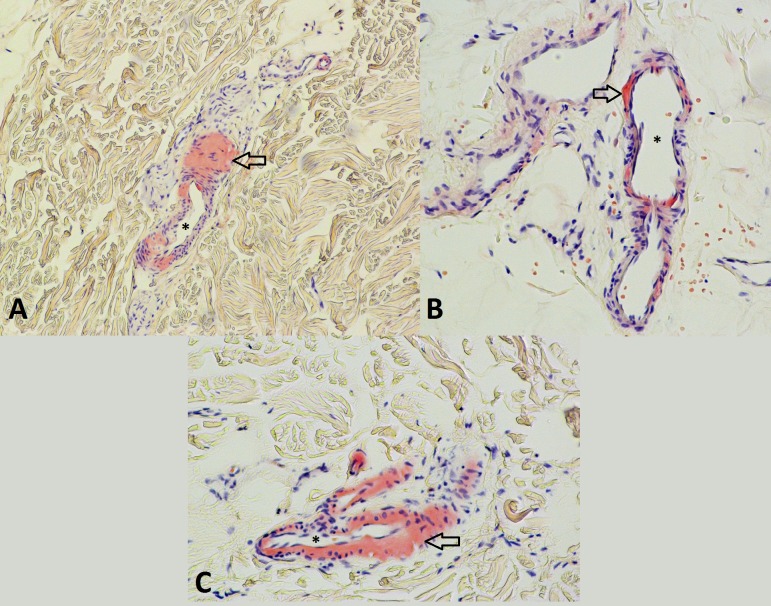
Abdominal fat pad biopsy Abdominal fat pad biopsy stained with Congo red showing amyloid deposits around the capillaries seen under optical microscopy. *Capillary lumen; arrows pointing to the amyloid deposits (pink).

These findings suggested the diagnosis of an infiltrative cardiomyopathy such as amyloidosis, hemochromatosis, or even sarcoidosis. Cardiovascular magnetic resonance (CMR) imaging scan was refused by the patient. Serum ferritin, transferrin saturation, and angiotensin converting enzyme levels were normal. The diagnosis of hemochromatosis and sarcoidosis became very unlikely, especially in the absence of any pulmonary or extra-pulmonary findings. The other infiltrative cardiomyopathies such as Fabry disease, Friedreich ataxia, Wegener disease, and many others, mainly occur in the younger population and were unlikely in this case. A fat pad biopsy was then performed which showed positive Congo red stain (Figure 3).

The serum and urine electrophoresis revealed an immunoglobulin G Lambda monoclonal spike along with high levels of free Lambda light chain (92 mg/L) and a decreased free Kappa to Lambda ratio of 0.1 (normal range is 0.26-1.65). At this point, multiple myeloma was highly suspected, but the bone survey failed to elicit any lytic lesions. Finally, a bone marrow biopsy was scheduled but the patient refused the procedure and left the hospital against medical advice. In conclusion, the patient was found to have light-chain (AL) amyloidosis secondary to an undiagnosed plasma cell dyscrasia, presenting as amyloid cardiomyopathy and autonomic dysfunction.

## Discussion

Amyloidosis is a general term that refers to the extracellular deposition of fibrils composed of low molecular weight subunits of a variety of proteins, many of which circulate as constituents of plasma. These soluble proteins undergo conformational changes and become insoluble by adopting a predominantly anti-parallel beta-pleated sheet configuration, leading to their deposition in various tissues [1]. There are several major forms of amyloidosis, such as AL amyloidosis, amyloid A (AA) amyloidosis, dialysis-related amyloidosis, heritable amyloidosis, and senile amyloidosis, among others. The clinical manifestations of amyloidosis are largely determined by (i) the type of precursor protein, (ii) the amount of amyloid deposition and most importantly, (iii) the tissue/organ involved [1]. This article focuses on amyloidosis that affects the heart muscle causing AC and discusses new diagnostic methods and current treatment options.

AC most commonly presents as heart failure with progressive dyspnea and edema. Other less common presenting symptoms are angina, syncope or pre-syncope [2]. It is worth noting that the presenting symptoms of cardiac amyloidosis in this patient were orthostatic hypotension, autonomic dysfunction, and frequent falls, with the lack of cardiac features such as angina, pulmonary edema, and lower extremity swelling. This is considered to be a rare and strange presentation of an advanced-stage amyloidosis with the involvement of the cardiac muscle. On physical examination, a variety of findings may be revealed in patients with cardiac amyloidosis such as increased jugular venous pressure, hepatomegaly due to liver congestion and profound peripheral edema. Thorough eyelid inspection can reveal periorbital purpura which is virtually a pathognomonic finding of cardiac amyloidosis if seen in a patient with heart failure) [2]. The most common abnormalities found on an ECG are low voltage and a pseudo-infarct pattern (poor R-wave progression in the precordial leads) in about 50% of the patients. Echocardiography constitutes the initial noninvasive test of choice to diagnose AC. The earliest findings are increased left ventricle (LV) wall thickness, generally symmetrical, along with diastolic dysfunction [3]. With the progression of the disease, the LV systolic function declines although the ventricular cavity size remains intact, the filling studies of the heart show a restrictive physiology. Right ventricular hypertrophy along with atrial enlargement and immobility are common features. Moreover, strain and strain rate imaging derived from speckle tracking typically show severe impairment of basal segments but preserved strain in the apical segments. This finding is rarely seen in other diseases and characterizes AC, unlike the previously described “granular or sparkling” appearance of the myocardium which reflects an increased echogenicity of the heart tissue lacking sensitivity and specificity for amyloidosis [3-4]. Recently, new studies showed an important role for CMR in providing strong evidence suggestive of AC, particularly when the distinctive pattern of global subendocardial late gadolinium enhancement is seen (sensitivity: 88%; specificity: 90%) [5]. Additionally, current systematic studies on radionuclide imaging investigated the role of a special tracer, the 99mTc-DPD, which appeared to localize to cardiac amyloid deposits very sensitively especially in patients with ATTR type. Indeed, these patients were identified with the aid of this tracer, even in asymptomatic stages, when echocardiography, serum cardiac biomarkers, and CMR were still normal, making this modality a potential screening and diagnostic tool [6]. While certain noninvasive tests such as echocardiography and CMR can highly suggest the presence of AC, a definitive diagnosis requires tissue confirmation either by demonstrating amyloid deposits on endomyocardial biopsy, or in patients with proper cardiac findings by detecting amyloid deposits on histologic examination of a biopsy from other tissues such as abdominal fat pad, rectum, or kidney [7]. The fibrils bind the Congo red stain and manifest the pathognomonic green birefringence under the polarized light on electron microscopy. Immunofluorescence microscopy can also be used to identify the type of protein subunit in many cases. Finally, a bone marrow biopsy should be a standard test in the initial workup of suspected amyloidosis, as it can demonstrate evidence of a plasma cell dyscrasia in more than 80% of patients and can show amyloid deposits in 60% of cases. In addition, if a very high plasma cell burden was found, this might suggest a co-existing multiple myeloma [4,7]. The differential diagnosis of cardiac amyloidosis includes various causes of infiltrative cardiovascular diseases, mainly sarcoidosis, Wegener disease, and hemochromatosis, among others [2,4,7]. The differentiation between these conditions can sometimes be done by noninvasive methods such as echocardiography and CMR, however, the need for tissue biopsy is frequently mandatory. A simultaneous systemic involvement of other organs such as kidneys and soft tissues causing nephrotic syndrome, macroglossia, and carpal tunnel syndrome, for example, can mask the cardiac presentation and delay the diagnosis and the initiation of therapy. Thus, physicians should look for cardiac involvement in any patient with suspected or established amyloidosis because it is the leading cause of mortality and morbidity in this disease. The prognosis of cardiac amyloidosis, despite treatment, remains poor and it is mainly dictated by the amyloid type, and the availability and response to therapy [2,4]. The treatment options are categorized into supportive therapy (i.e., modified heart-failure treatment), therapies that suppress the production of the respective amyloid fibril precursor protein (i.e., chemotherapy in AL amyloidosis), and the novel strategies that inhibit amyloid fibril formation or directly target the amyloid deposits. Cardiac transplantation is the last resort in carefully selected patients. Supportive treatment with the standard heart failure therapy may not be helpful or can even be deleterious, because beta-blockers, angiotensin-converting enzyme inhibitors, and angiotensin receptor blockers may worsen postural hypotension and/or renal function. These medications are poorly tolerated especially in the presence of restrictive cardiomyopathy where cardiac output is largely heart-rate-dependent. Also, the use of diuretics should be judicious, balancing between peripheral edema and renal impairment. However, when the use of diuretics is necessary, adding an α-agonist agent such as midodrine to help to maintain an adequate blood pressure, is suggested, especially if autonomic neuropathy is dominant [4,8]. Reducing amyloid fibril precursor protein production is a more aggressive option. The purpose of this therapy is reducing the amount of the respective amyloid fibril precursor protein by suppressing its source. For example, in AL amyloidosis, therapy is directed toward clonal plasma cells using either cyclical combination chemotherapy/immunomodulatory drugs (such as melphalan, thalidomide, and the newer agents bortezomib, lenalidomide, and pomalidomide), or high-dose therapy followed by autologous stem cell transplantation [8]. Another therapeutic option consists of inhibiting amyloid formation. The hypothesis behind this therapy resides on the pathophysiology of amyloidosis where amyloid fibril formation involves a massive conformational transformation of the respective precursor protein into a completely different form with predominant β-sheet structure. This conversion can be inhibited by stabilizing the fibril precursor protein and preventing its conformational changes. Among the drugs that might inhibit this transformation are diflunisal, tafamidis, and eprodisate [9]. Finally, targeting amyloid deposits by immunotherapy is a new concept involving passive immunotherapy to enhance clearance of amyloid by the human body itself, and it has proved successful in various experimental models. Anti-SAP monoclonal antibody is an encouraging model being studied currently in mice and is showing promising results [10].

The patient described in this report had biopsy-proven AC with typical electro- and echocardiographic findings, with peripheral neuropathy and autonomic dysfunction, secondary to a certain plasma cell dyscrasia with high concentrations of immunoglobulin G Lambda chains. Nonetheless, a definite diagnosis of the plasma cell dyscrasia could not be done because the patient refused further tests and procedures and was lost to follow-up.

## Conclusions

AC remains a challenging condition to diagnose and treat. Red flags that should raise suspicion include features of a multisystem disease, concentric LV hypertrophy on echocardiography with low voltage ECG, and a characteristic delayed enhancement pattern on CMR. Tissue biopsy remains the gold standard for diagnosis. Supportive treatment is limited to diuretic therapy when possible, and despite the developments in chemotherapy, the prognosis of patients with advanced cardiac involvement remains poor. Thereby, early recognition and diagnosis are essential for a better prognosis. In the end, a variety of novel specific therapies are currently on the horizon, with the ability to inhibit a new amyloid formation and enhance clearance of the existing fibrils.
